# Intensive Blood Pressure Control and Diabetes Mellitus Incidence for Patients with Impaired Fasting Glucose: A Secondary Analysis of SPRINT

**DOI:** 10.1155/2023/7533353

**Published:** 2023-03-16

**Authors:** Beiru Lin, Xiaochuan Liu, Sichen Yao, Zhigang Pan

**Affiliations:** ^1^Department of General Practice, Hainan West Central Hospital, Danzhou, Hainan, China; ^2^Department of General Practice, Zhongshan Hospital, Fudan University, Shanghai, China; ^3^Department of General Practice, Wujing Community Health Service Center, Shanghai, China

## Abstract

**Background:**

Previous studies indicated that intensive blood pressure (BP) control (systolic BP < 120 mm·Hg) compared with standard BP control (<140 mm·Hg) was associated with an increased risk of type 2 diabetes (T2D) and impaired fasting glucose (IFG) among hypertensive patients with normoglycemia. However, the impact of intensive BP control on the incidence of T2D for those with IFG is still unknown.

**Methods:**

This was a secondary analysis of the SPRINT (Systolic Blood Pressure Intervention Trial) of the study. We included participants with IFG at randomization, which was defined as fasting blood glucose (FBG) between 100 and 125 mg/dL. The primary outcome was incident T2D, defined as events of reaching FBG ≥ 126 mg/dL, participant self-report T2D at annual examination, or a record of hypoglycemic medications at follow-up. The secondary outcome was incident IFG reversion (IFGR), defined as the time to first FBG back to normoglycemia (<100 mg/dl) among participants without incident T2D. Cox proportional hazards models were used to compare the cumulative incidence of outcomes between the two BP control groups. Hazard ratios (HRs) with 95% confidence intervals (CIs) were calculated.

**Results:**

A total of 3310 participants were included in our primary outcome analysis (median age 67 years, 29% female). There were 293 participants who developed T2D among the intensive BP control group and 256 participants who developed T2D among the standard BP control group, resulting in 56.87 (50.36–63.39) versus 49.33 (43.29–55.37) events per 1000 person-years of treatment (HR 1.18 [95% CI, 1.00–1.40], *P*=0.052). After excluding 549 participants who developed T2D, 2761 participants were included in our secondary outcome analysis with 559 participants who developed IFGR among the intensive BP control group and 632 participants who developed IFGR among the standard BP control group, resulting in 141.20 (129.50–152.91) versus 158.20 (145.86,170.53) events per 1000 person-years of treatment (HR 0.9 [95% CI, 0.8–1.01], *P*=0.067).

**Conclusions:**

Our study found that in comparison to the standard BP control for hypertensive patients with IFG, intensive BP control was associated with a small increased risk of new-onset T2D, though it did not reach statistical significance. This kind of impact should be considered when implementing the strategy, especially for those with high risks of developing T2D. This trial is registered with NCT01206062.

## 1. Introduction

Hypertension and type 2 diabetes (T2D) are two common chronic conditions that often coexist in the same individual [1–3]. They have many shared risk factors such as obesity, diet, and insulin resistance. Due to shared risk factors and diverse classes of antihypertensive medications, a complex relationship has been reported between hypertension and T2D. Several studies have indicated that higher blood pressure is associated with an increased risk of T2D [4, 5]. Nearly 80% higher risk of T2D was observed with each 20 mm·Hg elevated systolic blood pressure (SBP) in a meta-analysis of cohort studies [5]. However, commonly used antihypertensive agents such as thiazide diuretics and *β*-blockers have been reported to be associated with increased risk of new-onset T2D, while angiotensin-converting enzyme inhibitors and angiotensin II receptor blockers were on the contrary [6–9]. Antihypertensive drug-drug interactions have been proposed as the underlying causes of the conflicting results.

With the publication of several positive clinical trials investigating the beneficial effect of intensive blood pressure (BP) control, a lower SBP target seems to be plausible among those who meet the criteria [10–12]. Strict SBP target often requires more antihypertensive agents, which would increase the risk of drug-drug interactions. A secondary analysis of SPRINT (Systolic Blood Pressure Intervention Trial) showed us that intensive BP control (SBP < 120 mm·Hg) compared with standard BP control (SBP < 140 mm·Hg) was associated with increased risk of T2D and impaired fasting glucose (IFG) among hypertensive patients with normoglycemia at the baseline [13]. Up to now, the impact of intensive BP control on the incidence of T2D in those with IFG at the baseline is still unknown. A secondary analysis of the SPRINT population might be able to fill this gap. In light of previous findings, our hypotheses in this study were that (1) intensive BP control was associated with increased risk of T2D for those with IFG at the baseline and (2) intensive BP control was associated with a lower rate of IFG reversion (IFGR) to normoglycemia.

## 2. Methods

### 2.1. Data Reproducibility Statement

SPRINT anonymized data are available at the National Heart, Lung, and Blood Institute (NHLBI) Biologic Specimen and Data Repository (https://biolincc.nhlbi.nih.gov/home/).

### 2.2. Study Design and Population

Our study was a secondary analysis of SPRINT, which is a multicenter randomized controlled trial that was conducted in the United States between November 2010 and March 2013. The major finding of the trial was that intensive BP treatment (SBP target <120 mm·Hg) compared with standard BP treatment (SBP target <140 mm·Hg) was more effective in preventing cardiovascular outcomes. Details of the trial have been reported elsewhere [14, 15]. Briefly, participants with screened SBP 130 to 180 mm·Hg and an increased risk of cardiovascular events were included. Participants were excluded if they had diabetes mellitus, severe heart failure, stroke, or dementia.

In this study, we further excluded participants who may have had diabetes mellitus at the baseline. Those with a missing record of blood glucose or with normoglycemia (<100 mg/dl), who had a fasting glucose ≥126 mg/dL, or who were on a glucose-lowering medication at randomization were also excluded. IFG was defined as fasting blood glucose (FBG) between 100 and 125 mg/dL.

This study was approved by the institutional review board (IRB) of each clinical facility, and all participants provided written informed consent.

### 2.3. Baseline Characteristics

Demographic and clinical data were collected through randomization. Medical histories were collected annually and included messages from hypoglycemic agents and self-reported T2D. BP was obtained by calculating the mean of 3 automated cuff readings with an automated device (Omron-HEM-907 XL) following standardized procedures. Laboratory data were collected at the baseline and at the 24 and 48 months or closeout visits. The value of blood glucose was considered missing if the sample was marked as nonfasting. Blood glucose was measured in serum using the hexokinase method on a Roche analyzer. Study covariates included age, gender, race, body mass index (BMI), number of antihypertensive drugs prescribed prior to randomization, and the Framingham risk score (FRS). Baseline estimated glomerular filtration rates (eGFR) were obtained by using the modification of diet in renal disease 4-component equation [16]. Previous cardiovascular disease (CVD) was defined as a history of clinical or subclinical CVD. Chronic kidney disease was defined as eGFR <60 mL/min/1.73 m^2^.

### 2.4. Outcome Definitions

The primary outcome of our study was incident T2D, defined as events of reaching FBG ≥ 126 mg/dL, participant self-report of T2D at annual examination, or a record of hypoglycemic medications at follow-up. The secondary outcome was incident IFGR, defined as the time to first FBG back to normoglycemia (<100 mg/dl) among participants without incident T2D.

### 2.5. Study Power Consideration

First of all, our study was a secondary analysis of SPRINT participants, so like many other secondary analyses, the power of our finding might be insufficient because the number of participants that fulfilled our study purpose was fixed. In our primary outcome analysis, 1,659 patients were allocated to the intensive BP control group and 1,651 patients were allocated to the standard BP control group. By using PASS 15.0 software, group sample sizes of 1,650 in group 1 and 1,650 in group 2 will achieve 57.151% power to detect a ratio of the group proportions of 1.18, which indicates that the power of our findings is insufficient. To achieve a power higher than 90%, the sample size needs to be about 7,000 (3,500 in each group).

### 2.6. Statistical Analysis

All statistical analyses were conducted in R version 3.6.2. where a *P* value <0.05 was considered statistically significant. Baseline characteristics were compared between participants with IFG randomized in the intensive BP control group and standard BP control group. Continuous variables were compared with the Wilcoxon rank sum test, and categorical variables were compared with Pearson's chi-squared test or Fisher's exact test. Cox proportional hazards models were used to compare the cumulative incidence of new-onset T2D between the intensive and standard BP control groups. For the comparison of the cumulative incidence of IFGR, we excluded participants who developed T2D after randomization. Hazard ratios (HRs) with 95% confidence intervals (CIs) were calculated, with the standard BP control group as the reference group. The follow-up time was censored at the end of the trial (August 20, 2015), upon death, failure to follow-up, or reaching the outcomes (T2D, IFGR). The proportional hazards assumptions were verified through checking Schoenfeld residuals. Subgroup analyses were conducted to test the interaction effect (treatment arm^*∗*^ subgroup) for our primary outcome among the following groups: age (≥75 and <75 years), race (black and nonblack), FRS (≥15% and <15%), number of antihypertensive drugs (≥2 and <2), gender (male and female), previous CVD (yes and no), SBP tertile at randomization (<132 mm·Hg; 132 to 145 mm·Hg; >145 mm·Hg), and baseline CKD (eGFR ≥60 and <60 mL/min/1.73 m^2^).

### 2.7. Sensitivity Analysis

First, we excluded participants who developed incident T2D within the first year of follow-up to explore the potential impact of reverse causality on our primary outcome. For the secondary outcome, we excluded participants who developed IFGR within 24 months of follow-up. Second, we excluded participants who withdrew their consent or failed to follow up after randomization to explore the potential impact of drop-out. Third, we compared the incidence of T2D and IFGR at different time points of follow-up between intensive and standard BP control groups.

## 3. Results

### 3.1. Characteristics of Study Participants

**Figure 1:**
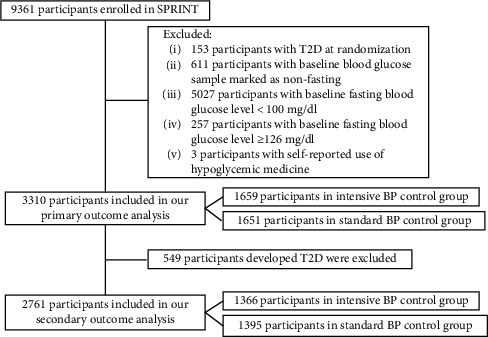
Study flowchart.

Figure 1 shows us the study flowchart. There were 9,361 participants enrolled in SPRINT. For the main analysis of incident T2D, we further excluded 6,051 participants: 153 participants had T2D at randomization; 611 participants had a baseline blood glucose sample marked as nonfasting; 5027 participants with the baseline blood glucose level <100 mg/dl; 257 participants had a baseline blood glucose level ≥126 mg/dl; and 3 participants with self-reported use of a hypoglycemic medicine. For the secondary analysis of the IFGR, we further excluded 549 participants who developed T2D after randomization. Baseline characteristics were well balanced between intensive and standard BP control groups, no matter in our primary analysis or in our secondary analysis (Table 1 and Supplemental Table s1). The median duration of follow-up was 3.22 years in our primary outcome analysis and 2.98 years in our secondary outcome analysis.

### 3.2. Impact of Intensive BP Control on Incident T2D

Out of the 3,310 participants included in our primary outcome analysis, 1,659 were allocated to the intensive BP control group and 1,651 were allocated to the standard BP control group (Figure 1). There were 293 participants who developed T2D among the intensive BP control group and 256 participants who developed T2D among the standard BP control group, resulting in 56.87 (50.36–63.39) versus 49.33 (43.29–55.37) events per 1,000 person-years of treatment (HR 1.18 [95% CI, 1.00–1.40], *P*=0.052, Table 2).

### 3.3. Impact of Intensive BP Control on Incident IFGR

After excluding 549 participants who developed T2D, 2,761 participants were included in our secondary outcome analysis (Figure 1). Of them, 1366 participants were allocated to the intensive BP control group. There were 559 participants who developed IFGR among the intensive BP control group and 632 participants who developed IFGR among the standard BP control group, resulting in 141.20 (129.50–152.91) versus 158.20 (145.86, 170.53) events per 1000 person-years of treatment, respectively (HR 0.9 [95% CI, 0.8–1.01], *P*=0.067, Table 2).

### 3.4. Subgroup Analysis of the Effect of Intensive BP Control on Incident T2D

**Figure 2:**
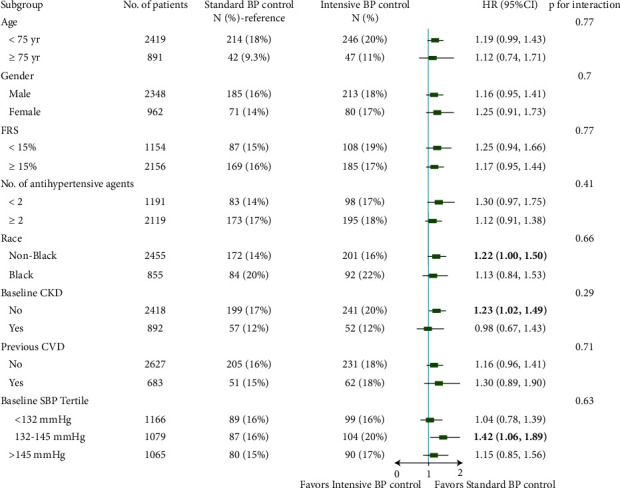
Risk of incident type 2 diabetes across subgroups between two treatment strategies. T2D: type 2 diabetes; BP: blood pressure; FRS: Framingham risk score; CKD: chronic kidney disease; CVD: cardiovascular disease; SBP: systolic blood pressure; HR: hazard ratio; CI: confidence interval; bold values mean statistical significance.

The interactive effect was tested between the treatment strategy and prespecified subgroups (Figure 2). Overall, no significant interactive effect was observed among all subgroups (*P* for interaction >0.05). Increased risk of T2D with intensive BP control was observed only among participants with eGFR ≥ 60 mL/min/1.73 m^2^ (HR 1.23 (95% CI, 1.02–1.49)), those whose SBP ranged from 132 to 145 mm·Hg at randomization (HR 1.42 (95% CI, 1.06–1.89)), and those with a self-reported race of nonblack (HR 1.22 [95% CI, 1.00–1.50]).

### 3.5. Sensitivity Analyses

After we excluded participants who developed incident T2D within the first year of follow-up and participants who developed IFGR within the 24 months of follow-up, the risks of incident T2D and IFGR were 1.16 (95% CI 0.98–1.38, *P*=0.09) and 0.89 (95% CI 0.73–1.09, *P*=0.05, Table s2), respectively, for the intensive BP control group. In addition, results were consistent as we excluded participants who failed to follow up or withdrew their consent (Table s3). As for the incidence of T2D and IFGR at different time points of follow-up between the two treatment groups, no significant differences were observed except for the incidence of T2D at 24 months (5.3% versus 7.2%, *P*=0.033, Table s4).

## 4. Discussion

Our study is an extension of the findings revealed by Roumie et al. [13] who found that intensive BP control compared with standard BP control was associated with increased risk of T2D and IFG among participants with normoglycemia at randomization. Through our study, risk of T2D was also found to be increased among participants with IFG at randomization treated with intensive BP control, though it did not reach statistical significance. It is worth noting that the confidence interval for the risk of T2D was also wide in the study conducted by Roumie et al. Subgroup analysis indicated intensive BP control was associated with increased risk of T2D among participants with eGFR ≥ 60 mL/min, those with SBP ranging from 132 to 145 mm·Hg at randomization, and those with self-reported race of nonblack. As for the capability of reverting the progression of IFG, intensive BP control was not associated with a higher percentage of participants with IFGR. In contrast, it might have a negative impact on IFGR.

Recently, several large randomized controlled trials have shown us that intensive BP control to a lower SBP target can reduce the risk of cardiovascular events for hypertensive patients with high cardiovascular risk. SPRINT indicated that intensive SBP control of <120 mm·Hg can achieve impressive cardiovascular benefits in comparison to standard SBP control of <140 mm·Hg [11]. The BP measurement method of SPRINT (automated Omron-HEM-907 XL) was different from other BP control trials (office BP). Although the procedures for BP measurement in SPRINT were consistent with other trials, some questioned whether the BP readings may be misinterpreted in SPRINT due to the absence of staff in the room, resulting in lower BP values than those obtained in other trials or clinical practice. However, the benefit observed in SPRINT was similar to that in other trials. STEP (Strategy of Blood Pressure Intervention in the Elderly Hypertensive Patients) found that intensive SBP treatment of <130 mm·Hg resulted in 26% lower incidence of cardiovascular events than standard SBP treatment of <150 mm·Hg in Chinese hypertensive patients [10]. Secondary analysis of participants who received standard glycemic therapy in the ACCORD (Action to Control Cardiovascular Risk in Diabetes Blood Pressure trial) identified benefits similar to those seen in SPRINT treated with intensive BP control [12, 17]. In light of these findings, the recommended SBP target has a tendency to be lower than previously recommended in many hypertension management guidelines [18–20]. Achieving a lower SBP target is administered with caution in daily practice. Concerns over adopting this intensive therapy mainly arise from its adverse events. However, the impact of intensive BP control on the metabolism of blood glucose should also be considered. There was a 19% higher risk of T2D and a 17% higher risk of IFG among those who received intensive BP control with normoglycemia at randomization [13]. To our surprise, there was only 18% higher risk of T2D among those who received intensive BP control with IFG at randomization since IFG is commonly considered as a prediabetes condition. This observation needs to be investigated in the future. Physicians should have a thorough discussion with patients about risks and benefits of pursuing intensive BP target, especially for those with high risks of developing T2D [21].

As for the reasons why intensive BP control can have an impact on the metabolism of blood glucose is still unknown. On one hand, different classes of antihypertensive drugs have been reported to be associated with the risk of T2D [6–9, 22]. Due to the limited sample size and observational nature of previous studies, a causal relationship cannot be achieved. On the other hand, the interaction of different classes of antihypertensive drugs was also likely to be associated with the risk of T2D. In SPRINT, the use of multiple antihypertensive drugs among both the intensive and standard BP control groups makes it difficult to investigate the relationship between drug-drug interaction and the risk of T2D. A well-designed meta-analysis of individual patient data from clinical trials or a mendelian randomization study might be the promised ways for future research studies to answer the remaining questions.

### 4.1. Limitations

Some limitations should be considered when interpreting the results of our study. First, this was a secondary analysis of SPRINT, so the power was not enough to detect differences in risk of T2D between the two treatment groups, as suggested in our study power consideration. The impact may be stronger in our subgroup analysis. For future research studies, pooled individual data of several finished trials may have sufficient power to detect differences between the two groups regarding the incidence of T2D. Although baseline characteristics were well balanced between the two treatment groups, residual confounders such as insulin levels and markers of insulin resistance may have an impact on our results. Second, the definitions of IFG and IFGR were based on a single fasting glucose test at the baseline and time-updated measures of glycemic control. We cannot exclude the possibility of differing risks of T2D if IFG had been based on HbA1c levels or an oral glucose tolerance test; however, this kind of data is not available in SPRINT. To test the robustness of our findings, we excluded participants with IFGR that happened within 24 months after randomization, and results were consistent as shown in our primary analysis. Third, the definition of incident T2D included participants with self-reported T2D at the annual examination or a record of hypoglycemic medications at follow-up. Because the diagnosis was not adjudicated by a physician, this raised concerns over misreporting. Finally, as we know the median follow-up time of SPRINT was only about 3 years, the overall risk of T2D might change with a longer duration of follow-up.

## 5. Conclusion

Our study found that, in comparison to standard BP control for hypertensive patients with IFG, intensive BP control was associated with a small increased risk of new-onset T2D, though it did not reach statistical significance. This kind of impact should be considered when implementing the strategy, especially for those with high risks of developing T2D.

## Figures and Tables

**Table 1:** Baseline characteristics of study participants.

Characteristics	Overall	Standard BP control	Intensive BP control	*p* value
	*N* = 3310	*N* = 1651	*N* = 1659	
Age	67 (61, 75)	67 (61, 75)	67 (61, 75)	0.8
Gender (female)	962 (29%)	494 (30%)	468 (28%)	0.3
BMI (kg/m^^2^)	29.9 (26.9, 33.7)	29.8 (27.0, 33.5)	29.9 (26.7, 34.0)	0.9
SBP (mmHg)	138 (129, 148)	138 (129, 148)	137 (129, 148)	0.5
DBP (mmHg)	78 (70, 86)	77 (70, 86)	78 (70, 85)	0.6
SBP tertile				
<132 mm·Hg	1,166 (35%)	560 (34%)	606 (37%)	0.2
132−145 mm·Hg	1,079 (33%)	558 (34%)	521 (31%)	
>145 mm·Hg	1,065 (32%)	533 (32%)	532 (32%)	
FRS (≥15%)	2,156 (65%)	1,075 (65%)	1,081 (65%)	>0.9
Race (black)	855 (26%)	429 (26%)	426 (26%)	0.8
Number of antihypertensive agents				
<2	1,191 (36%)	604 (37%)	587 (35%)	0.5
≥2	2,119 (64%)	1,047 (63%)	1,072 (65%)	
Aspirin	1,774 (54%)	867 (53%)	907 (55%)	0.2
Smoking status				
Never smoked	1,428 (43%)	722 (44%)	706 (43%)	0.8
Former smoker	1,515 (46%)	741 (45%)	774 (47%)	
Current smoker	365 (11%)	187 (11%)	178 (11%)	
Missing data	2 (<0.1%)	1 (<0.1%)	1 (<0.1%)	
Baseline CKD	892 (27%)	457 (28%)	435 (26%)	0.3
Previous CVD	683 (21%)	337 (20%)	346 (21%)	0.8
eGFR (ml/min/1.73 m^2^)	72 (59, 85)	71 (58, 85)	73 (59, 85)	0.3
Serum creatinine (mg/dL)	1.01 (0.87, 1.20)	1.03 (0.86, 1.21)	1.00 (0.87, 1.20)	0.4
CHR (mg/dL)	184 (159, 212)	185 (160, 214)	184 (158, 211)	0.1
GLUR (mg/dL)	106 (102, 112)	106 (102, 111)	106 (102, 112)	0.12
HDL (mg/dL)	48 (41, 57)	48 (41, 57)	48 (41, 57)	0.4
TRR (mg/dL)	115 (83, 163)	114 (82, 162)	115 (84, 164)	0.9
Urine albumin-to-creatinine ratio (mg/g)	9 (6, 21)	9 (5, 22)	10 (6, 20)	0.7

Data were expressed as median (interquartile range) or number (percentage); BP: blood pressure; BMI: body mass index; SBP: systolic blood pressure; DBP: diastolic blood pressure; FRS: Framingham risk score; CKD: chronic kidney disease; CVD: cardiovascular disease; eGFR: estimated glomerular filtration rates; CHR: cholesterol; GLUR: glucose; HDL: high density lipoprotein cholesterol direct; TRR: triglycerides; to convert the values for creatinine to micromoles per liter, multiply by 88.4. To convert the values for cholesterol to millimoles per liter, multiply by 0.02586. To convert the values for triglycerides to millimoles per liter, multiply by 0.01129. To convert the values for glucose to millimoles per liter, multiply by 0.05551. Race and ethnic group were self-reported. BMI is the weight in kilograms divided by the square of the height in meters.

**Table 2:** Incident outcome events by two treatment strategies.

Incident T2D	Standard BP control *N* = 1651	Intensive BP control *N* = 1659	*p* value
Median duration of follow-up	3.22 years		
No. of events	256 (16%)	293 (18%)	0.1
Person time years	5189.34	5151.73	
HR (95% CI)	Reference	1.18 (1.00–1.40)	0.052
Incidence rate per 1000 person-years of treatment strategy (95% CI)	49.33 (43.29, 55.37)	56.87 (50.36, 63.39)	

Incident IFGR	*N* = 1395	*N* = 1366	

Median duration of follow-up	2.98 years		
No. of events	632 (45%)	559 (41%)	0.02
Person time years	3994.99	3958.85	
HR (95% CI)	Reference	0.9 (0.8–1.01)	0.067
Incidence rate per 1000 person-years of treatment strategy (95% CI)	158.20 (145.86, 170.53)	141.20 (129.50, 152.91)	

T2D: type 2 diabetes; IFGR: impaired fasting glucose reversion; BP: blood pressure; HR: hazard ratio; CI: confidence interval.

## Data Availability

SPRINT anonymized data are available at the National Heart, Lung and Blood Institute (NHLBI) as the biological specimen and Data Repository (https://biolincc.nhlbi.nih.gov/home/).

## Supplementary Materials

Supplementary Materials Table s1: baseline characteristics of study participants included in our secondary outcome analysis. Table s2: incident outcome events by two treatment strategies after excluding participants who developed incident events within the first year or 24 months of follow-up. Table s3: incident outcome events by two treatment strategies after excluding participants who were lost to follow-up or withdrew their consent. Table s4: incidence of outcome events and blood glucose levels at different time points of follow-up.

## References

[1] Reaven G. M. (1991). Insulin resistance, hyperinsulinemia, hypertriglyceridemia, and hypertension. Parallels between human disease and rodent models. *Diabetes Care*.

[2] Reaven G. M., Lithell H., Landsberg L. (1996). Hypertension and associated metabolic abnormalities--the role of insulin resistance and the sympathoadrenal system. *New England Journal of Medicine*.

[3] Reaven G. M. (1991). Relationship between insulin resistance and hypertension. *Diabetes Care*.

[4] Fletcher A., Amery A., Birkenhager W. (1991). Risks and benefits in the trial of the European working party on high blood pressure in the elderly. *Journal of Hypertension*.

[5] Emdin C. A., Anderson S. G., Woodward M., Rahimi K. (2015). Usual blood pressure and risk of new-onset diabetes: evidence from 4.1 million adults and a meta-analysis of prospective studies. *Journal of the American College of Cardiology*.

[6] Elliott W. J., Meyer P. M. (2007). Incident diabetes in clinical trials of antihypertensive drugs: a network meta-analysis. *The Lancet*.

[7] Abuissa H., Jones P. G., Marso S. P., O’Keefe J. H. (2005). Angiotensin-converting enzyme inhibitors or angiotensin receptor blockers for prevention of type 2 diabetes: a meta-analysis of randomized clinical trials. *Journal of the American College of Cardiology*.

[8] Gillespie E. L., White C. M., Kardas M., Lindberg M., Coleman C. I. (2005). The impact of ACE inhibitors or angiotensin II type 1 receptor blockers on the development of new-onset type 2 diabetes. *Diabetes Care*.

[9] Jandeleit-Dahm K. A., Tikellis C., Reid C. M., Johnston C. I., Cooper M. E. (2005). Why blockade of the renin-angiotensin system reduces the incidence of new-onset diabetes. *Journal of Hypertension*.

[10] Zhang W., Zhang S., Deng Y. (2021). Trial of intensive blood-pressure control in older patients with hypertension. *New England Journal of Medicine*.

[11] The Sprint Research Group, Lewis C. E., Fine L. J. (2021). Final report of a trial of intensive versus standard blood-pressure control. *New England Journal of Medicine*.

[12] Beddhu S., Chertow G. M., Greene T. (2018). Effects of intensive systolic blood pressure lowering on cardiovascular events and mortality in patients with type 2 diabetes mellitus on standard glycemic control and in those without diabetes mellitus: reconciling results from ACCORD BP and SPRINT. *Journal of American Heart Association*.

[13] Roumie C. L., Hung A. M., Russell G. B. (2020). Blood pressure control and the association with diabetes mellitus incidence: results from SPRINT randomized trial. *Hypertension*.

[14] Ambrosius W. T., Sink K. M., Foy C. G. (2014). The design and rationale of a multicenter clinical trial comparing two strategies for control of systolic blood pressure: the Systolic Blood Pressure Intervention Trial (SPRINT). *Clinical Trials*.

[15] The Sprint Research Group, Wright J. T., Williamson J. D. (2015). A randomized trial of intensive versus standard blood-pressure control. *New England Journal of Medicine*.

[16] Levey A. S., Bosch J. P., Lewis J. B., Greene T., Rogers N., Roth D. (1999). A more accurate method to estimate glomerular filtration rate from serum creatinine: a new prediction equation. *Annals of Internal Medicine*.

[17] Buckley L. F., Dixon D. L., Wohlford G. F., Wijesinghe D. S., Baker W. L., Van Tassell B. W. (2017). Intensive versus standard blood pressure control in SPRINT-eligible participants of ACCORD-BP. *Diabetes Care*.

[18] Joint Committee for Guideline Revision (2019). 2018 Chinese guidelines for prevention and treatment of hypertension-A report of the revision committee of Chinese guidelines for prevention and treatment of hypertension. *Journal of Geriatric Cardiology*.

[19] Whelton P. K., Carey R. M., Aronow W. S. (2018). 2017 ACC/AHA/AAPA/ABC/ACPM/AGS/APhA/ASH/ASPC/NMA/PCNA guideline for the prevention, detection, evaluation, and management of high blood pressure in adults A report of the American college of cardiology/American heart association task force on clinical practice guidelines. *Journal of the American College of Cardiology*.

[20] Williams B., Mancia G., Spiering W. (2018). 2018 ESC/ESH Guidelines for the management of arterial hypertension. *European Heart Journal*.

[21] Nazarzadeh M., Rahimi K. (2020). Intensive blood pressure lowering and risk of diabetes: friend or foe?. *Hypertension*.

[22] Nazarzadeh M., Bidel Z., Canoy D. (2021). Blood pressure lowering and risk of new-onset type 2 diabetes: an individual participant data meta-analysis. *The Lancet*.

