# Supplementary material for: Intensive Blood Pressure Control and Diabetes Mellitus Incidence for Patients with Impaired Fasting Glucose: A Secondary Analysis of SPRINT
Source: Int J Hypertens. 2023 Mar 16;2023:7533353. doi: 10.1155/2023/7533353 (PMC10036175; doi:10.1155/2023/7533353)
Supplement: Supplementary Materials — Table s1: baseline characteristics of study participants included in our secondary outcome analysis. Table s2: incident outcome events by two treatment strategies after excluding participants who developed incident events within the first year or 24 months of follow-up. Table s3: incident outcome events by two treatment strategies after excluding participants who were lost to follow-up or withdrew their consent. Table s4: incidence of outcome events and blood glucose levels at different time points of follow-up. [file 7533353.f1.pdf]

Online supplemental material

Table s1: Baseline characteristics of study participants included in our secondary outcome analysis

Characteristics	Overall<br>N= 2761	Standard BP control<br>N= 1395	Intensive BP control<br>N= 1366	p-value
Age	68 (61, 76)	68 (61, 76)	68 (61, 76)	>0.9
Gender (Female)	811 (29%)	423 (30%)	388 (28%)	0.3
BMI, (kg/m^^{2})	29.5 (26.7, 33.2)	29.4 (26.8, 33.0)	29.5 (26.5, 33.4)	0.9
SBP, mmHg	138 (129, 148)	138 (129, 148)	137 (129, 148)	0.4
DBP, mmHg	77 (69, 85)	77 (69, 85)	77 (69, 85)	0.7
SBP Tertile				0.11
<132 mmHg	978 (35%)	471 (34%)	507 (37%)	
132−145mmHg	888 (32%)	471 (34%)	417 (31%)	
>145mmHg	895 (32%)	453 (32%)	442 (32%)	
FRS (≥15%)	1,802 (65%)	906 (65%)	896 (66%)	0.7
Race (Black)	679 (25%)	345 (25%)	334 (24%)	0.9
Number of antihypertensive agents				0.4
<2	1,010 (37%)	521 (37%)	489 (36%)	
≥2	1,751 (63%)	874 (63%)	877 (64%)	
Aspirin	1,485 (54%)	738 (53%)	747 (55%)	0.3
Smoking status				0.9

Never smoked	1,197 (43%)	608 (44%)	589 (43%)	
Former smoker	1,276 (46%)	636 (46%)	640 (47%)	
Current smoker	286 (10%)	150 (11%)	136 (10.0%)	
Missing data	2 (<0.1%)	1 (<0.1%)	1 (<0.1%)	
Baseline CKD	783 (28%)	400 (29%)	383 (28%)	0.7
Previous CVD	570 (21%)	286 (21%)	284 (21%)	0.9
eGFR, mL/min/1.73m^2	71 (58, 84)	70 (58, 84)	72 (58, 85)	0.2
Serum creatinine, mg/dL	1.02 (0.87, 1.21)	1.03 (0.86, 1.22)	1.01 (0.87, 1.21)	0.4
CHR, mg/dL	184 (158, 212)	185 (160, 214)	183 (157, 210)	0.15
GLUR, mg/dL	105 (102, 110)	105 (102, 110)	105 (102, 110)	0.5
HDL, mg/dL	49 (42, 58)	49 (42, 58)	49 (41, 58)	0.3
TRR, mg/dL	113 (81, 158)	112 (81, 158)	113 (82, 156)	0.8
Urine albumin-to-creatinine ratio, mg/g	10 (6, 22)	10 (5, 22)	10 (6, 21)	0.4

Data were expressed as median (interquartile range) or number(percentage); BP: blood pressure; BMI: body mass index; SBP: systolic blood pressure; DBP: diastolic blood pressure; FRS: Framingham risk score; CKD: chronic kidney disease; CVD: cardiovascular disease; eGFR: estimated glomerular filtration rates; CHR: Cholesterol; GLUR: Glucose; HDL: high density lipoprotein cholesterol direct; TRR: Triglycerides; To convert the values for creatinine to micromoles per liter, multiply by 88.4. To convert the values for cholesterol to millimoles per liter, multiply by 0.02586. To convert the values for triglycerides to millimoles per liter, multiply by 0.01129. To convert the values for glucose to millimoles per liter, multiply by 0.05551. Race and ethnic group were self-reported. BMI is the weight in kilograms divided by the square of the height in meters.

Table s2 Incident outcome events by two treatment strategies after excluding participants who developed incident events within the first year or 24 months of follow-up

Incident T2D	Standard BP control N = 1582	Intensive BP control N = 1594	p-value
No. of events	244 (15%)	274 (17%)	0.2
HR (95% CI)	Reference	1.16(0.98-1.38)	0.09
Incident IFGR	N = 979	N = 992	
No. of events	216 (22%)	185 (19%)	0.06
HR (95% CI)	Reference	0.89(0.73, 1.09)	0.05

Abbreviations: T2D: type 2 diabetes; IFGR: impaired fasting glucose reversion; BP: blood pressure; HR: hazard ratio; CI: confidence interval;

Table s3 Incident outcome events by two treatment strategies after excluding participants who loss to follow-up or withdraw their consent

Incident T2D	Standard BP control N = 1564	Intensive BP control N = 1583	p-value
No. of events	256 (16%)	288 (18%)	0.2
HR (95% CI)	Reference	1.16(0.98-1.37)	0.09
Incident IFGR	N = 1308	N = 1295	
No. of events	627 (48%)	557 (43%)	0.012
HR (95% CI)	Reference	0.90(0.80-1.00)	0.058

Abbreviations: T2D: type 2 diabetes; IFGR: impaired fasting glucose reversion; BP: blood pressure; HR: hazard ratio; CI: confidence interval;

Table s4 Incidence of outcome events and blood glucose levels at different time points of follow-up

Incident T2D	No. of participants	Overall<br>N = 3310	Standard BP control<br>N = 1651	Intensive BP control<br>N = 1659	p-value
12m	3,310	62 (1.9%)	24 (1.5%)	38 (2.3%)	0.076
		N = 3248	N = 1627	N = 1621	
24m	3,248	203 (6.2%)	87 (5.3%)	116 (7.2%)	0.033
		N = 3045	N = 1540	N = 1,505	
36m	3,045	69 (2.3%)	32 (2.1%)	37 (2.5%)	0.5
		N = 2976	N = 1508	N = 1468	
Closeout	2,976	215 (7.2%)	113 (7.5%)	102 (6.9%)	0.6
**Incident IFGR**		N = 2761	N = 1395	N = 1366	
24m	2,761	790 (29%)	416 (30%)	374 (27%)	0.2
		N = 1971	N = 979	N = 992	
48m	1,971	172 (8.7%)	91 (9.3%)	81 (8.2%)	0.4
		N = 1799	N = 888	N = 911	
Closeout	1,799	229 (13%)	125 (14%)	104 (11%)	0.091
**Glucose level (median, IQR)**					
24m	2,402	103 (97, 109)	102 (96, 109)	103 (98, 110)	0.003
48m	569	103 (98, 109)	103 (98, 109)	104 (97, 109)	>0.9
Closeout	937	105 (99, 113)	104 (98, 112)	105 (99, 113)	0.14

Abbreviations: BP: blood pressure; m: month;
